# Design of the Quality of Life in Motion (QLIM) study: a randomized controlled trial to evaluate the effectiveness and cost-effectiveness of a combined physical exercise and psychosocial training program to improve physical fitness in children with cancer

**DOI:** 10.1186/1471-2407-10-624

**Published:** 2010-11-11

**Authors:** Katja I Braam, Elisabeth M van Dijk, Margreet A Veening, Marc B Bierings, Johannes HM Merks, Martha A Grootenhuis, Mai JM Chinapaw, Gerben Sinnema, Tim Takken, Jaap Huisman, Gertjan JL Kaspers, Eline van Dulmen-den Broeder

**Affiliations:** 1 VU University Medical Center, department of Paediatrics and Division of Oncology-Haematology, Amsterdam, the Netherlands; 2 VU University Medical Center, department of Medical Psychology, Amsterdam, the Netherlands; 3 Wilhelmina Children's Hospital/UMC Utrecht, Paediatric Haematology/oncology, Utrecht, the Netherlands; 4 Emma Children's Hospital/Academic Medical Centre, department of Paediatric Oncology, Amsterdam, the Netherlands; 5 Emma Children's Hospital/Academic Medical Centre, Psychosocial Department, Amsterdam, the Netherlands; 6 EMGO Institute for Health and Care Research, department of Public and Occupational Health, VU University Medical Center, Amsterdam, the Netherlands; 7 Wilhelmina Children's Hospital/UMC Utrecht, department of Medical Psychology, Utrecht, the Netherlands; 8 Wilhelmina Children's Hospital/UMC Utrecht, Child Development and Exercise Center, Utrecht, the Netherlands

## Abstract

**Background:**

Childhood cancer and its treatment have considerable impact on a child's physical and mental wellbeing. Especially long-term administration of chemotherapy and/or radiotherapy impairs physical fitness both during and after therapy, when children often present with muscle weakness and/or low cardiorespiratory fitness. Physical exercise can improve these two elements of physical fitness, but the positive effects of physical exercise might be further increased when a child's wellbeing is simultaneously enhanced by psychosocial training. Feeling better may increase the willingness and motivation to engage in sports activities. Therefore, this multi-centre study evaluates the short and long-term changes in physical fitness of a child with a childhood malignancy, using a combined physical exercise and psychosocial intervention program, implemented during or shortly after treatment. Also examined is whether positive effects on physical fitness reduce inactivity-related adverse health problems, improve quality of life, and are cost-effective.

**Methods:**

This multi-centre randomized controlled trial compares a combined physical and psychosocial intervention program for children with cancer, with care as usual (controls). Children with cancer (aged 8-18 years) treated with chemotherapy and/or radiotherapy, and who are no longer than 1 year post-treatment, are eligible for participation. A total of 100 children are being recruited from the paediatric oncology/haematology departments of three Dutch university medical centres. Patients are stratified according to pubertal stage (girls: age ≤10 or >10 years; boys: ≤11 or >11 years), type of malignancy (haematological or solid tumour), and moment of inclusion into the study (during or after treatment), and are randomly assigned to the intervention or control group.

**Discussion:**

Childhood cancer patients undergoing long-term cancer therapy may benefit from a combined physical exercise and psychosocial intervention program since it may maintain or enhance their physical fitness and increase their quality of life. However, the feasibility, patient need, and effectiveness of such a program should be established before the program can be implemented as part of standard care.

**Trial registration number:**

NTR1531 (The Netherlands National Trial Register)

## Background

Advances in diagnosis and treatment of childhood cancer have dramatically increased long-term survival. Consequently, the number of childhood cancer survivors (CCS) is growing and it is now evident that the disease and its treatment can significantly impair long-term health [1]. Adverse effects of a paediatric malignancy and its treatment are both frequent and diverse, and may emerge years after the completion of therapy. One study reported that about 75% of CCS has at least one late adverse health effect after a median follow-up of 17 years [1]. Therefore, paediatric oncologists and others involved in the care of childhood cancer patients need to focus not only on survival, but also on the quality of survival.

Impaired physical fitness (e.g. reduced cardiorespiratory function and/or decreased muscle strength) has been reported both during and after childhood cancer treatment [2-6]. Since physical fitness represents the functional status of many body functions involved in the performance of daily physical activities and/or physical exercise, physical fitness is considered an important health marker. Physical inactivity with subsequent muscle atrophy and reduced strength is probably the most prominent cause of this reduced state of physical fitness [6-9]. Again, this low physical fitness may lead to fatigue, obesity and a poor skeletal and/or mental health [7,9-14]. In turn, these factors may further reduce physical activity and physical fitness making these health outcomes a self-perpetuating condition if the cycle is not reversed.

All of these health outcomes (separately or simultaneously) may negatively impact both short and long-term health and observed health-related quality of life (HrQOL) [12,14]. Therefore, prevention of inactivity-related health problems by increasing physical fitness, both during and after treatment, is essential.

Rehabilitation programs in adult cancer patients, including physical exercise and psychosocial support, report positive effects on physical fitness and HrQOL [15,16]. However, such programs are not available for children with cancer. For them it may be even more important to maintain physical fitness and psychosocial health, since childhood cancer occurs in a crucial period of life. During childhood essential physiological and psychological changes take place, and the basis for adult behaviour, lifestyle and health status is established [17,18]. No or little participation in physical activity over a longer period of time may have significant implications for future physical activity performance, physical and psychosocial health outcomes, and ultimately HrQOL [19,20].

Studies describing physical exercise and its effects on muscle strength and physical fitness during and after treatment of childhood cancer are scarce, are performed in small study groups, and/or do not include a psychosocial support program to increase wellbeing, self-belief and compliance with the intervention [21-24]. The available studies show that it is safe for childhood cancer patients to engage in exercise interventions. However, high drop-out rates occur in physical exercise studies among cancer patients, leading to low powered or unbalanced data [25,26]. This was seen, for example, in a Dutch study testing a 12-week exercise training program (combining aerobic and strength exercises) in 9 childhood acute lymphoblastic leukaemia survivors (aged 6-18 years), in continuous remission and at least 3 years after diagnosis [27]. The 5 drop-outs (44%) and their parents found the 12-week sessions too demanding and/or difficult to combine with school attendance and other activities [27]. In addition, it appeared that parental support and patient self-belief are important factors related to compliance.

Based on these experiences, for the present study the 12-week exercise training program was modified. The revised intervention program now combines a more gradual increase of exercise intensity with a psychosocial intervention aimed to enhance patient wellbeing, patient self-belief and parental support. The short and long-term effectiveness of this combined physical and psychosocial intervention, implemented during or shortly after cancer treatment in a large group of childhood cancer survivors, is evaluated in a randomized controlled trial (RCT). In this study physical fitness is the primary outcome; also examined is whether positive effects on physical fitness reduce inactivity-related adverse health problems, improve HrQOL, and are cost-effective. The design of this RCT is described below.

## Methods

This study is part of a larger program of the Dutch Cancer Society. This larger Alpe d'HuZes Cancer Rehabilitation Research Program (A-CaRe) consists of four RCTs evaluating exercise intervention in different groups of cancer patients; these four studies use similar methodologies and outcome measures. An overview of the A-CaRe program has been described elsewhere [28]. Quality of Life in Motion (QLIM) is the only cancer and sport study of A-CaRe in the childhood cancer population.

This RCT among children with cancer, compares the primary and secondary outcome measures of the study between an intervention and a care-as-usual control group. In addition, cost utility and cost effectiveness is evaluated.

The intervention training program of QLIM consists of two elements. First the physical intervention, a structured, individualized and supervised physical exercise program of moderate to high intensity. Secondly the psychosocial intervention, which is an individualized structured program to enhance socio-emotional functioning and coping with disease-related effects. Improved wellbeing is expected to increase willingness and motivation to engage in sports activities and, as a result, enhance the efficacy of the exercise program.

### Study sample

Eligible participants are aged between 8 and 18 years at the time of inclusion into the study, diagnosed with any type of childhood malignancy, treated with chemotherapy and/or radiotherapy, and are still receiving treatment, or are no longer than 12 months off treatment. Patients requiring a bone marrow transplantation and/or growth hormone therapy are excluded. In addition, patients who depend on a wheelchair, who are not able to 'ride a bike', read, write, self-reflect, and/or follow instructions are also excluded from this study.

### Recruitment and randomisation

**Figure 1:**
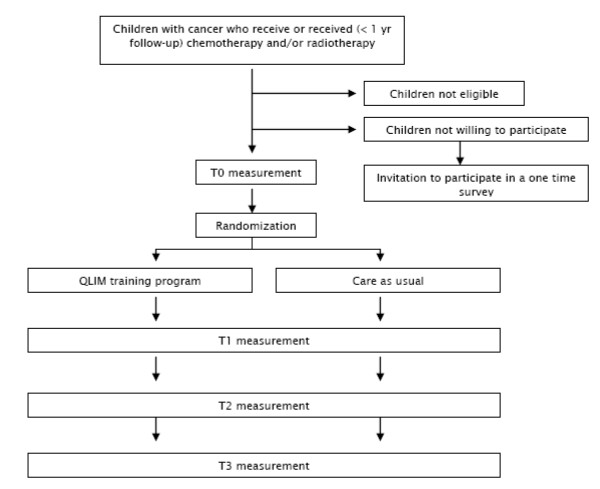
**Flowchart of the Quality of Life in Motion (QLIM) study**.

Hundred children are being recruited from the department of Paediatric Oncology and Haematology of the VU University Medical Center (VUmc) in Amsterdam, the Wilhelmina Children's Hospital UMC in Utrecht (WKZ/UMCU), and the Emma Children's Hospital/Academic Medical Center (EKZ/AMC) in Amsterdam. Patient inclusion starts after approval of the Medical Ethics Committee (number 2008/208). The patients and their parents individually receive written and verbal information about the study, an informed consent form, and an addressed return envelope (Figure 1). Written informed consent is obtained from the parents or legal guardian of each patient, and also separately from each patient aged 12 years and older.

Baseline measurements (performance tests and questionnaires) occur as soon as the clinical condition of the patient enables him/her to participate in and complete the intervention program. After these baseline measurements the patient is randomised to either the intervention or the control group after being stratified according to type of malignancy (haematological cancer or solid tumour), pubertal stage (girls: age ≤10 or >10 years; boys: ≤11 or >11 years), and moment of inclusion into the study (during or after treatment). For those patients randomised to the intervention group the QLIM intervention starts within 2 weeks after baseline measurements. The control group receives care as usual.

### The QLIM intervention

The intervention arm of the study consists of a combined physical and a psychosocial intervention.

#### Physical intervention

Since childhood cancer and its treatment are associated with a loss of both aerobic capacity and muscle strength, the 12-week physical intervention includes a combination of both cardiorespiratory and muscle strength training (twice a week for 45 min) at a physical therapy sports centre near the child's home. Training is performed individually, under the supervision of an experienced paediatric physical therapist. Prior to the start of the intervention period all therapists receive the intervention protocol and personal instructions; during the intervention period a site visit is made to guarantee uniformity of the intervention between therapists.

**Figure 2:**
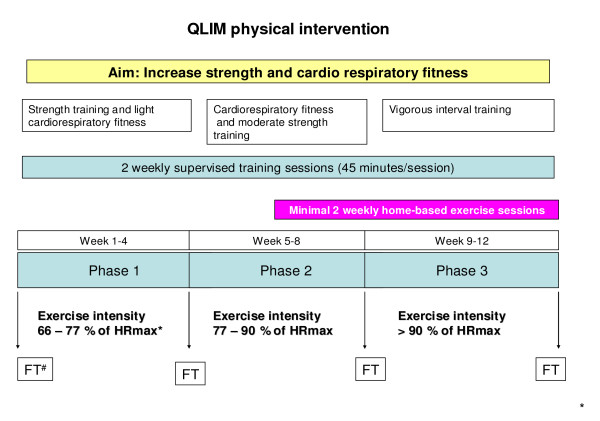
**Details of the Quality of Life in Motion (QLIM) physical intervention**. HFmax, maximum heart rate. ^# ^FT, Field Tests: Modified Shuttle Walk Test; Steep Ramp Test; 10 × 5 meter sprint test; 30 second repetition maximum for sit-ups, push-ups, head and leg raises; 60-second repetition maximum for squats.

The physical intervention consists of 3 phases of one month each (Figure 2). The primary goal of the 1^st ^phase is to increase muscle strength; the 2^nd ^phase aims to increase cardiorespiratory fitness; and the 3^rd ^phase aims to further increase both cardiorespiratory fitness and muscle strength through interval training. To improve compliance with the program, the sessions are of varying content. The intensity of the sessions gradually increases throughout the study. The intensity of the cardiopulmonary training is assessed by heart rate monitoring during the training session, whereas strength is assessed by the number of repetitions made per time period. Progression of physical fitness is monitored by field tests [29,30] at the start and at the end of each phase by the therapists in the local sports centres. The aim of these field tests is to assess training progress for both the participant and their trainer.

In week 6 to 12, children are instructed to perform five additional exercises at home (at least twice a week). These exercises have an increasing intensity and frequency to enhance strength, flexibility and aerobic fitness [31]. This aims to further increase the training effects and to introduce physical exercise into their daily routine to encourage an active lifestyle after the intervention has ended. This home-based training is accompanied by music designed and timed for the duration of the exercises.

The performed level of activity, the number of repetitions achieved and the date are recorded in the physical training workbook. Patients randomised to the intervention group are also allowed to participate in additional sports activities and are stimulated to participate in school sports.

#### Psychosocial intervention

The psychosocial intervention is specifically developed for the QLIM study. The theoretical framework and exercises are based on clinical care for childhood cancer patients and on a cognitive-behavioural based group training that is used in clinical care for chronically ill children at the VUmc (not published). Regular cognitive-behavioural intervention techniques have been modified for this specific intervention program. The intervention is provided by a trained paediatric psychologist in the treatment centre of the child. An independent psychologist checks the quality and similarity between therapists in random site visits to the QLIM sessions.

The psychosocial intervention includes six child sessions of 60 min each (once every 2 weeks) and two parent sessions (Table 1). The first element of the psychosocial intervention is psycho-education on disease-related topics. For adolescents, sexual orientation, and growth and developmental topics are added. Subsequently cognitive-behavioural components are incorporated in the program to intensify awareness of hidden feelings, thoughts, behaviour and their consequences, and to enhance coping strategies. The content of the program, psycho-educational information and assignments are standardized and compiled in an instruction manual.

**Table 1:** Details of the Quality of Life in Motion (QLIM) psychosocial intervention.

Psychosocial training modules	**Includes 6 child sessions and 2 parent sessions**.
Session 1 (1 h)	In the *first *module the focus is on increasing self-awareness and belief in one's own competency. During an exercise, the patient is stimulated and supported to express positive things about him/herself.
Session 2 (1 h)	The *second *module focuses on feelings, thoughts, and behaviour. The central themes are the four basic feelings of a human being: fear, anger, happiness and sadness.
Session 3 (1 h)	In the *third *module coping with (non) disease-related difficult situations is the main topic. Also, training in relaxation exercises is given.
Session 4 (1 h)	The *fourth *module focuses on social contacts with peers. Does the disease influence their social relationships? For the older age group, sexuality and relationships are also addressed.
Session 5 (1 h)	The *fifth *module focuses on possible changes in the family after the diagnosis of cancer, i.e. changes in relations with siblings and parents, and separation-individuation problems.
Session 6 (1 h)	The *sixth *session deals with the future and how to incorporate physical exercise in daily life from now onwards.
Parent session 1 (1 h)	The first parent session focuses on the principles of the program to increase parental support, in order to improve compliance and endurance.
Parent session 2 (1 h)	The second and last parent session focuses on evaluation of the total program. Parents and therapists describe the achieved goals and the changes observed during the program.

To enhance the effects of the psychosocial intervention for the individual patient, the manual is divided into several modules with different topics. All topics are discussed with each patient. The number and intensity of assignments for each patient are individually tailored. The tailored program is made according to baseline information from the parents and patient, and according to the clinical evaluation of the paediatric psychologist during the intervention. Because parent support has a strong impact on a child's behaviour, two parent sessions are included to increase endurance and compliance with the QLIM intervention. The last parent session evaluates the child's functioning before and during the intervention, and the observed changes.

All patients in the intervention group are invited for a half-day booster session 3-6 months after completion of the 12-week intervention period. This booster session involves a combined group sports clinic and a psychosocial session during which participants discuss what they have learned during both elements of the QLIM intervention.

#### Control group

Controls are not restricted to perform activities such as school or leisure sports. Although usual care can not be standardized, it will not involve systematic exercise training or psychosocial support. However, if the patients have any physical or psychosocial problems they have access to regular health care (e.g. a physiotherapist or medical psychologist).

### Study outcomes

#### General

The primary outcome of the present study is physical fitness (cardiorespiratory fitness and muscle strength). Secondary outcomes are fatigue, body composition, bone mineral density (BMD), daily physical activity, depression, HrQOL, self-perception and behaviour. Additionally, compliance and satisfaction with the intervention are evaluated by self-report; compliance is also evaluated via the therapist's report. Also assessed are potentially moderating variables (including pre-illnesses, lifestyle, health, exercise-related attitudes, beliefs and motivations) of both the child and parents.

Physical fitness is assessed by measuring both the cardiorespiratory fitness and muscle strength. BMD and fat mass are determined by a DEXA scan (lumbar spine, total body and hip) as gold standard, combined with bone markers in blood and body mass index (Table 2). Daily physical activity is assessed by wearing an Actical 2.1 accelerometer from Wednesday to Saturday on the left hip, and by keeping a physical activity diary. Fatigue, quality of life, depression, self-perception and behavioural problems are assessed by questionnaires [32-36]. In addition, the cost effectiveness of the study is determined by the use of a per month cost diary. It gathers information concerning visits to health care givers, work and school participation of both the parents and the child and performed social activities, To facilitate comparison of results across A-CaRe studies, the outcome measures used in these four RCTs have been standardized as far as possible. A detailed description of the secondary outcome measures common to all four RCTs has been reported elsewhere [28].

**Table 2:** Details of the primary outcome measures and the measurement tools used.

Primary outcome measures	Measurements
Cardiorespiratory fitness	Lode Corival bicycle ergometry with paediatric options; LODE BV, Groningen, the Netherlands. Steep Ramp Test [35] VO_2 _peak performance test

Muscle strength	CITEC hand-held dynamometer; C.I.T. Technics BV, Haren, the Netherlands

**Secondary outcome measures**	**Measurements**

Fatigue	PedsQL Multidimensional Fatigue Scale Acute Version (self-report and parent-proxy report) [30]

Body composition	Dual Energy X-ray Absorptiometry (DEXA) scan (Hologic QDR 4500); Hologic Bedford, USA. Body Mass Index Blood: calcium, phosphate, magnesium, carboxy-terminal collagen crosslinks (CTX), parathyroid hormone (PTH), serum amino-terminal procollagen 1 extension peptide (P1NP), insulin-like growth factors 1 (IGF-1), 25-hydroxy-vitamin D (25 OH vit D)

Daily physical activity levels	Physical activity accelerometer: Actical 2.1; Respironics, Mini Mitter, Oregon USA

Depression	Children's Depression Inventory (CDI) [32]

Quality of life	PedsQL 4.0 Generic Score Scale (self-report and parent-proxy report) [30] PedsQL 3.0 Cancer Module (self-report and parent-proxy report) [30]

Self-perception	Self-Perception Profile for Children (CBSK) [31] Self-Perception Profile for Adolescents (CBSA) [33]

Behavioural problems	Child Behavior Checklist (CBCL) [34] Youth Self-Report (YSR) [34]

Cost data	Monthly cost diary

**Other study outcomes**	**Measurements**

Compliance	Self-report Attendance checklist for training sessions

Satisfaction with the intervention	Questionnaire 11-items

Clinical data	Medical record

Moderating variables	Questionnaire addressing pre-illness lifestyle, current attitudes towards and beliefs about exercise, physical activity patterns.

All patients perform fitness tests, use an accelerometer, and complete a battery of questionnaires prior to randomization (T0), after 14-20 weeks (T1), and at 12-months follow-up (T3). At T2 (6-9 months from baseline) only the data from the questionnaires and the accelerometer data are collected.

##### Cardiorespiratory fitness

Cardiorespiratory fitness is assessed by two VO_2 peak _performance tests: the Steep Ramp Test and the cardiopulmonary exercise test according to the Godfrey protocol, both on an electronically braked Lode Corival bicycle ergometer (Lode B.V., Groningen, the Netherlands). The test protocol includes one minute of rest and three minutes of cycling without resistance before the start of the Steep Ramp Test [37]. The workload at the beginning of the test is 25 watts which increases every 10 seconds by an additional 25 watts until exhaustion. The Steep Ramp test is followed by three minutes of cycling without resistance and two minutes of no movement. Following these five minutes the second test the cardiopulmonary exercise test, is introduced in which the workload increases by 10, 15 or 20 watts every minute depending on the child's body height, as described in the Godfrey protocol [38]. The patients are instructed to cycle at a speed of 60-80 rotations per minute until he/she stops because of volitional exhaustion. The patients breath through a facemask connected to a calibrated metabolic cart. Breath-by-breath minute ventilation, oxygen consumption, carbon dioxide production, and the respiratory exchange ratio are calculated from conventional equations. Heart rate is measured continuously during the aerobic exercise test by a bipolar electrocardiogram or heart rate monitor, as is done for the oxygen percentage by saturation measurement. VO_2 peak _is calculated as the average value over the last 30 seconds before subjective exhaustion.

Prior to the aerobic exercise test, pulmonary function is assessed at rest by measuring the forced air expiratory volume in 1 s and the forced vital capacity using a (portable) spirometer. The best out of 3 forced expiratory flow-volumes (in upright position) are recorded. Pulmonary function can be reliably assessed in young children [39].

##### Muscle strength

Muscle strength of the proximal and distal muscles in the upper and lower extremities is measured by the calibrated Citec hand-held dynamometer (CIT Technics, Groningen, the Netherlands) at the right and the left side of the body. Three consecutive measurements are performed using the 'break method', in which the examiner gradually overcomes the muscle force and stops at the moment the extremity gives way [40]. The highest value of three repetitions is registered. In the upper extremity, grip strength and strength of the shoulder abductors and the elbow extensors are measured. In the lower extremity, the muscular strength of the hip flexors and the knee and dorsal foot extensors are measured. All within-subject tests are performed by the same assessor using the same hand-held dynamometer to prevent inter-instrument and inter-observer bias [41]. The same assessors perform the tests in the intervention and in the control group.

### Characteristics of non-responders

To compare the responders and non-responders, patients and parents not wishing to participate in the study, are asked to fill in a one-time survey regarding their reasons for non-participation, their daily physical activity, their general quality of life (PedsQL 4.0 Generic Score Scale self-report and parent-proxy report) and behavioural problems (CBCL for parents, YSR for patients aged 11 years and older). In addition, specific disease and treatment information is obtained from the patient's medical records.

### Power calculation

Power calculations were performed on expected physical fitness changes. It is expected that the physical fitness of patients randomised to care as usual will deteriorate due to disease and treatment-related factors causing physical inactivity. However, it is conceivable that patients randomized to the control group are triggered to increase their normal physical activity level due to the information provided about the study. Therefore, we hypothesize that controls will maintain, rather than increase, their physical fitness during treatment since structured exercise will not be offered. In the intervention study arm, however, we expect an improvement of at least 20% in physical fitness based on data from San Juan et al. [21]. These authors found that the mean VO_2 peak _(ml/lkg/min) at baseline increased from 24.3 ml/lkg/min (SD 5.9) to 30.2 ± 6.2 SD following the intervention [21]. Therefore, in order to detect a 20% difference between the intervention and the control group [standard deviation difference (Cohen's effect size) 0.8] with a power of 80% and an alpha of 0.05 (2-sided test) at least 26 children per group are required according to the nomogram for sample size calculations [42]. The plan is to recruit 100 patients into the study to allow for an attrition of about 40% (i.e. patients who discontinue participation in the study, including failure to complete follow-up assessments). Those who discontinue participation in the intervention group, but complete the follow-up assessments, will be included in the analysis on an intention-to-treat basis. However, due to the modifications made to the 12-week exercise training program after the pilot study, we expect attrition to be significantly lower in the present study, which will significantly increase the power of the study. For intervention participants with an attrition of at least 75% a per-protocol analysis will be performed with data of the first measurement from baseline and, secondly. with data from the final measurement.

### Statistical analysis

Longitudinal multi-level regression analysis is used to assess changes over time in study participants. In these, for between-group differences, the Longitudinal Generalised Estimating Equations (GEE) analysis is used. In addition, participants with at least one post-intervention assessment are analyzed according to the intention-to-treat principle. Scores on the self-report measures of fatigue, quality of life, depression, self-perception and behavioural problems are calculated according to published scoring algorithms. Finally, we investigate whether compliance with the intervention is significantly associated with changes over time in physical condition, muscle strength, HrQOL and fatigue, and also determine possible determinants of effectiveness. Test results are considered significant for p-values < 0.05.

### Cost-effectiveness analysis

This study also includes a cost-effectiveness and cost-utility analysis to determine and compare the difference in total cancer-related costs of the treatment group, and the control group. In this analysis both direct and indirect costs are taken into account. The cost-utility ratio presents the additional costs of the intervention per quality-adjusted life years (QALY); details are published in [28].

## Discussion

This paper presents the design and methods of the QLIM study (A-CaRe study 4), an RCT evaluating the effect of a combined physical and psychosocial intervention on the physical fitness of children with cancer. For different chronic diseases exercise-based rehabilitation is recommended. Although this is also the case for cancer, high-quality evidence for its effectiveness is lacking. The QLIM intervention is based on the theoretical framework and results from adult cancer rehabilitation studies and practice, and on pilot data from studies in children who survived cancer. The strength of this study is that the QLIM offers children an intervention combining both physical and psychosocial exercises. Cancer generally diminishes a child's capability to perform normal daily activities. The QLIM aims to reverse the negative self-perpetuating circle of becoming more and more inactive. The psychosocial intervention is added to improve wellbeing and thereby enhance efficacy of the exercise program. In addition to the child sessions, this psychosocial intervention includes two parent sessions to enhance parental support, which might also increase program compliance.

Due to the relatively low incidence of paediatric cancer and a mean age at diagnosis of 4 years, the number of eligible participants is relatively small. To enrol a sufficient number of patients almost all types of cancer are included, resulting in a heterogeneous study population. To ensure an equal distribution among the intervention and control group we therefore stratify for pubertal stage, type of cancer, and phase of treatment.

## Competing interests

The authors declare that they have no competing interests.

## Authors' contributions

All authors contributed to the study design (with the exception of MAG and JHM who reinforced the QLIM study at a later stage). KIB and EMD are responsible for patient recruitment and data collection. KIB coordinates the logistics of the physical intervention and EMD, who developed the psychosocial intervention, coordinates the psychosocial intervention and performs the psychosocial interventions at the VU University Medical Center. Both KIB and EMD wrote the manuscript and contributed equally; GJK, JHU and TTA are principal investigators. GJK is the overall project leader. JHU leads the psychosocial content of the study; TTA developed the physical intervention and is the paediatric exercise physiology leader of the study; MAV, MBB and JHM are paediatric oncologists and clinically responsible for, respectively, the VU University Medical Center, the Wilhelmina Children's Hospital/University Medical Center Utrecht and the Emma Children's Hospital/Academic Medical Centre, Amsterdam; GSI and MAG are responsible for the psychosocial interventions in, respectively, the Wilhelmina Children's Hospital/University Medical Center Utrecht and the Emma Children's Hospital/Academic Medical Centre, Amsterdam; MCP directs the four A-CaRe trials; EVD was responsible for writing the grant proposal for funding from the Dutch Cancer Society. All authors contributed to and approved the final manuscript.

## Pre-publication history

The pre-publication history for this paper can be accessed here:

http://www.biomedcentral.com/1471-2407/10/624/prepub
